# High density lipoprotein promoting proliferation and migration of type II alveolar epithelial cells during inflammation state

**DOI:** 10.1186/s12944-017-0482-x

**Published:** 2017-05-18

**Authors:** Zhongji Yu, Jingru Jin, Yuhui Wang, Jian Sun

**Affiliations:** 1 The Fourth Affiliated Hospital of Nanchang University, Nanchang, 330003 China; 2 People’s Hospital of Shangrao City, Shangrao, 334000 China; 3 The Institute of Cardiovascular Sciences and Institute of Systems Biomedicine, School of Basic Medical Sciences, and Key Laboratory of Molecular Cardiovascular Sciences of Ministry of Education, Peking University Health Science Center, Beijing, 100191 China

**Keywords:** High density lipoprotein, Type II Alveolar epithelial cells, Inflammation, Proliferation, Migration

## Abstract

**Background:**

To investigate the effect and mechanism of high density lipoprotein (HDL) on type II alveolar epithelial cells during inflammation state.

**Methods:**

The original generation of type II alveolar epithelial cells were separated in rats and treated with PBS/LPS/HDL/HDL + LPS. To observe the proliferation and migration of type II alveolar epithelial cells with bromodeoxyuridine(BrdU) assay, transwell assay and wound healing experiments. In addition, western blot detected the expression of TP-binding cassette transporter A1 (ABCA1), cystic fibrosis transmembrane conductance regulator (CFTR) and the phosphorylation of AKT/extracellular signal-regulated kinase(ERK)/mitogen-activated protein kinase(MAPK). Enzyme-linked immunosorbent assay (ELISA) tested the secretion of tumor necrosis factor a(TNF-a)/interleukin 1a(IL-1a)/IL-6.

**Results:**

HDL promoted the proliferation (↑17%, *p* < 0.001 HDL+ LPS vs. LPS) and migration (wounding healing: ↑93%, *p* < 0.001 HDL+ LPS vs. LPS; transwell migration: ↑154%, *p* < 0.001 HDL+ LPS vs. LPS) of type II alveolar epithelial cells. Furthermore, HDL increased the phosphorylation of MAPK, but not AKT/ERK. And HDL decreased the secretion of TNF-a (↓46%, *p* < 0.01 HDL+ LPS vs. LPS) and IL-1a (↓45%, *p* < 0.001 HDL+ LPS vs. LPS), but not IL-6. In addition, HDL up-regulated the expression of ABCAI (↑99%, *p* < 0.001 HDL vs. CON) and down-regulated the expression of CFTR (↓25%, *p* < 0.05 HDL vs. CON) in type II alveolar epithelial cells.

**Conclusions:**

HDL increases the phosphorylation of MAPK, which promotes the proliferation and migration of type II alveolar epithelial cells**.** And it decreased the secretion of TNF-a/IL-1a and the expression of CFTR. All these suggest that HDL plays an important role in anti-inflammatory effect in inflammation state of lung.

## Background

Pulmonary alveoli, composed of type I and type II alveolar epithelial cells, are the sites for gas exchange between the blood and the atmosphere. Type II alveolar epithelial cells take charge of the production and turnover of pulmonary surfactant, the protein–lipid mixture that serves to lessen the surface tension in the alveolus permitting for lung ventilation smoothly [1]. Type I alveolar epithelial cells do not proliferate, when injured they are replaced by type II alveolar epithelial cells that transdifferentiate into type I alveolar epithelial cells.

HDL mediate reverse cholesterol transport out of cells. Many studies have shown that HDL has atheroprotective functions [2]. In addition, the role of anti-inflammation, anti-oxidation and anti-apoptotic, were also tested [3]. These functions are increasingly being recognized to be active in the pulmonary system [4]. These indicated that HDL and apoA-I have protective effects in pathophysiological states.

Some studies shown that HDL inhibit the secretion of inflammation cytokines and against endotoxin [5]. Some studies tested that HDL inhibit cytokine-induced expression of adhesion molecules [6]. Some studies revealed that HDL plays a role of anti-inflammation in lung as it is the major source of vitamin E for type II pneumocytes and alveolar surfactant is supplemented with vitamin E during its assembly in type II pneumocytes [7].

To better understand the effect of HDL on type II alveolar epithelial cells during inflammation state, this study observed the proliferation and migration of type II alveolar epithelial cells stimulated by lipopolysaccharide (LPS) and HDL. We also examined the signaling pathways of proliferation and migration, including PI3K/Akt, extracellular signal-regulated kinase (Erk1/2) and mitogen-activated protein kinase (p38MAPK).

## Methods

### Ethics statement

Healthy subjects were recruited in our study. Each participant provided written informed consent. The study protocol was approved by the Institutional Review Board of the Fourth Affiliated Hospital of Nanchang University, China.

### Cell culture and material

Type II alveolar epithelial cells were isolated as described previously [8] and cultured in Dulbecco’s modified Eagle’s medium (DMEM; GIBCO, UK) supplemented with 10% fetal bovine serum. Cells were grown on 0.1% (*w*/*v*) gelatin-coated culture ware and then cultured in a humidified incubator at 37 °C with an atmosphere of 5% CO_2_.

Antibodies for western blotting against ABCA1 and b-actin were from Abcam, USA. Antibody against Phospho-Akt, phospho-ERK1/2 and phospho-p38 MAPK were from Cell Signaling Technology, USA. Anti-ERK1/2 antibody, anti-p38MAPK antibody, anti-Akt antibody were purchased from Santa Cruz Biotechnology (Santa Cruz, CA). HRP-goat-anti-rabbit IgG and HRP-goat-anti-mouse IgG were purchased from MBL (Nagoya, Japan)BrdU proliferation ELISA kit was from Roche Applied Science, Germany.

### Isolation of lipoproteins

Plasma was obtained by centrifugation from peripheral blood of fresh fasting healthy subjects. This part was approved by the local ethics committee. HDL (d = 1.063–1.210 g/ml) was isolated by sequential ultracentrifugation as described elsewhere [9]. Resulting preparations of lipoproteins were dialyzed against of phosphate-buffered saline (PBS) (pH = 7.4) containing 1 mM EDTA and 100 μM diethylenetriamine pentaacetic acid (DTPA) (Sigma, USA), sterilized with 0.22-μm filter, stored away from light at 4 °C and used within 1 months. HDL concentration was determined by immunoturbidimetrical detection of apolipoprotein A-I (Roche Diagnostic).

### BrdU proliferation assay

Type II alveolar epithelial cells (5 × 10^3^ cells/well) were seeded in 96-well plates with Dulbecco’s Modified Eagle’s Medium and 10% fetal bovine serum and cultured overnight. And then, Type II alveolar epithelial cells were treated with HDL (100 mg/ml apoA-I concentration), LPS (1μg/ml), LPS + HDL or Control for 48 h. The cells were labeled with 20 ml/well of BrdU labeling solution as described previously [10], and then incubated with 200 ml/well of FixDenat. After incubated with 100 ml/well of Anti-BrdU-POD working solution for 90 min and washed 3 times, substrate solution (TMB) was added and the absorbance of each well was read at 450 nm with an ELISA plate reader (model 550, BioRad, USA).

### Wound healing

Type II alveolar epithelial cells were plated with Dulbecco’s Modified Eagle’s Medium and 10% fetal bovine serum in 24-well plate (5× 10^5^/ml cells/ well). And then, type II alveolar epithelial cells were serum deprived and scratched, treated with HDL (100 mg/ml apoA-I concentration), LPS (1μg/ml), LPS + HDL or Control. 18 h later Type II alveolar epithelial cells were fixed with methanol, stained with hematoxylin-eosin stain, and viewed under an inverted microscope (Nikon, Japan).

### Transwell experiments

Quantitative migration assays with type II alveolar epithelial cells were performed using a modified Boyden chamber (Minicell, Millipore, USA) as described previously [11]. The Transwell insert was placed back to the 24-well plate and the lower chamber was filled with 0.6 ml of Dulbecco’s Modified Eagle’s Medium and 5% bovine serum. Type II alveolar epithelial cells (5000 cells/well) in Dulbecco’s Modified Eagle’s Medium were plated into the upper chamber. HDL (100 mg/ml apoA-I concentration), LPS (1μg/ml), LPS + HDL or Control was individually added to the upper chamber. After 8 h incubation, all nonmigrated cells were removed from the upper face with a cotton swab, and migrated cells were fixed and stained with hematoxylin-eosin stain. Cell migration was quantified by counting the number of stained cells per in 10 random fields photographed for each chamber.

### Type II alveolar epithelial cells -ECM adhesion assay

Type II alveolar epithelial cells pretreated with HDL or LPS were incubated in wells coated with Matrigel Membrane Matrix [12]. Wells were coated with 20 ml of 0.1 mg/ml Matrigel Membrane Matrix (Vigorous Biotechnology Beijing Co., China) in PBS at room temperature and air dry in 96-well plates. The type II alveolar epithelial cells were serum deprived and pretreated with N-HDL or LPS for 24 h before plating into each well. The plates were blocked with 20 ml 2% BSA at 37uC for 1 h, and then washed with PBS. The pretreated type II alveolar epithelial cells in DMEM with 0.1% BSA were plated in each well at a density of 8*10^4^ cells/well for 1 h at 37uC. Thereafter, non-adherent cells were removed by washing with PBS 3 times and 40 mg/well of MTT was added for 4 h at 37uC. After discarding MTT and adding 200 ml DMSO, and measuring the absorption at 570 nm (model 550, BioRad, USA).

### ELISA analysis of TNF-a、IL-1a and IL-6

Cells were seeded onto six-well plates and then treated with HDL, LPS in serum-free DMEM for 6 h. Supernatants were collected, centrifuged (10 min, 1500 g) at room temperature and stored at −80 °C until use. Concentrations of TNF-a, IL-1a and IL-6 were determined from cell culture supernatants.

### Western blot analysis

After treatments, cells were washed with PBS and lysed in RIPA buffer. Cell debris was removed by centrifugation (12,000 *g* for 15 min), and protein concentrations were quantified using the Coomassie brilliant blue method. Equal amounts of total protein were separated by SDS-PAGE and then blotted onto nitrocellulose membranes. The membranes were blocked in 5% non-fat dry milk for 1 h and then incubated with primary antibodies for 4 h followed by the appropriate HRP-conjugated secondary antibody for 2 h at room temperature.

### Statistical analysis

All experiments were repeated at least three times. Analysis of variance was performed using GraphPad Prism (San Diego, CA, USA). One-way ANOVA was conducted to compare overall differences among multiple groups. Post-hoc comparisons were performed using Tukey’s or Bonferroni’s test. A *p*-value of <0.05 was considered statistically significant. Data are expressed as the mean ± SD unless stated otherwise.

## Results

### HDL stimulates Type II alveolar epithelial cells proliferation

**Fig. 1:**
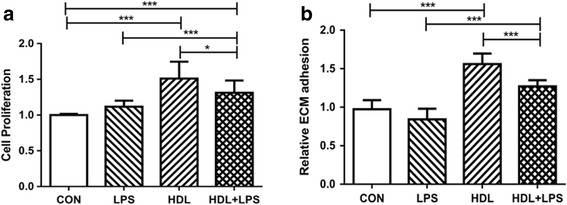
HDL Promoting Proliferation and adhesion to ECM of Type II Alveolar Epithelial Cell. **a** Type II Alveolar Epithelial were treated with control, HDL, LPS and HDL + LPS, and cell proliferation was measured using BrdU assay (*n* = 3). **b** Type II Alveolar Epithelial were treated with control, HDL, LPS and HDL + LPS for 24 h, and relative cell adhesion to ECM was determined after 1 hour incubation (*n* = 3). (*P < 0.05; ***P < 0.001)

BrdU tested the proliferation of type II alveolar epithelial cells induced by HDL. The incubation of HDL (100 mg/ml apoA-I) led to 51% increase in relative cell number compared to control and 35% increase in relative cell number compared to LPS. The incubation of HDL+ LPS led to 17% increase in relative cell number compared to LPS but 13% decrease in relative cell number compared to HDL. (Figure 1a, *n*= 3, *p*<0.001 comparing HDL vs. con; *p*<0.001 comparing HDL+ LPS vs. LPS).

### HDL stimulates Type II alveolar epithelial cells migration

**Fig. 2:**
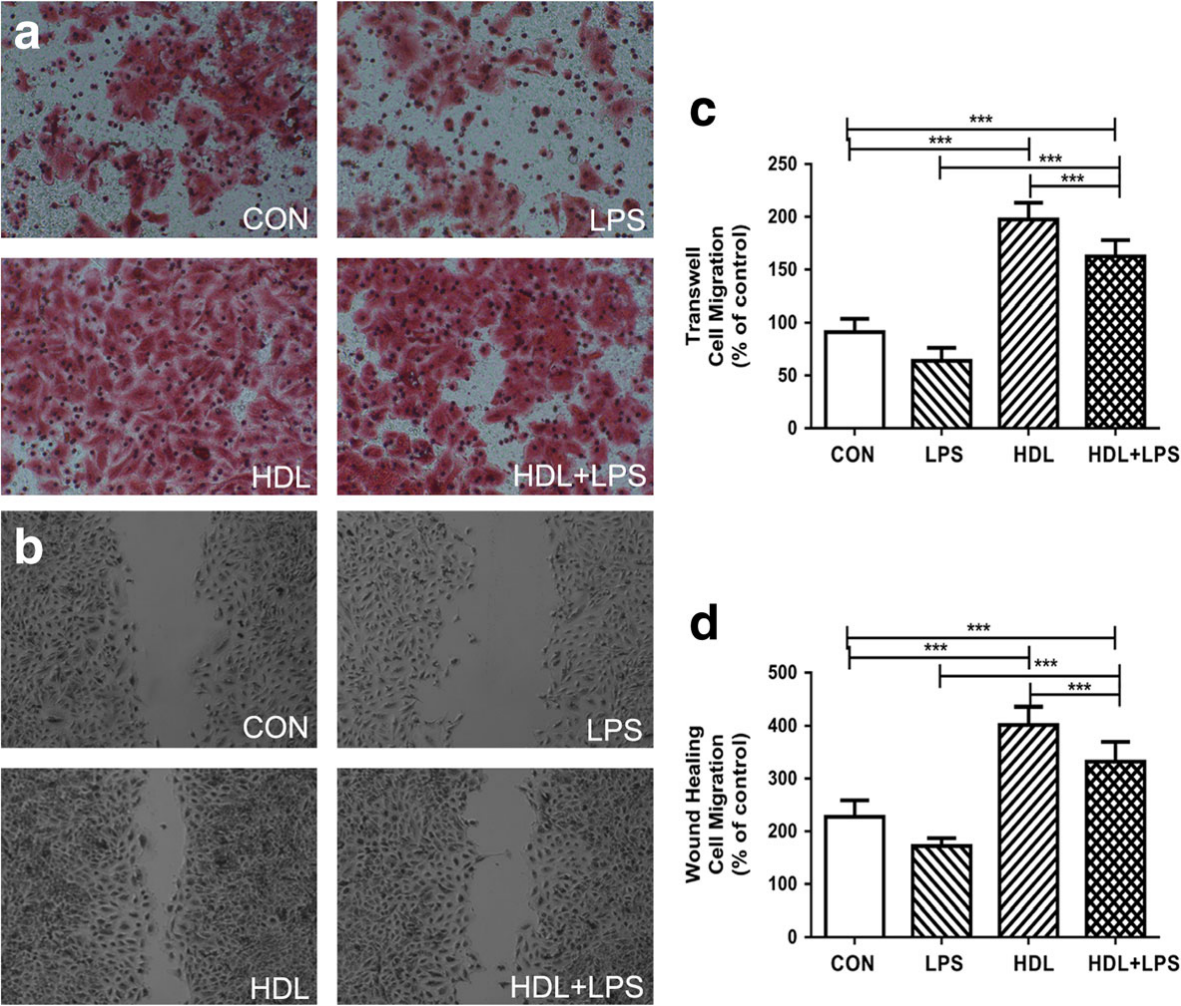
HDL Promoting Migration of Type II Alveolar Epithelial Cell. **a** Type II Alveolar Epithelial were treated with control, HDL, LPS and HDL + LPS, and transwell migration was evaluated after 8 h. **c** Quantification values are expressed as the mean ± SD (*n* = 3). **b** Type II Alveolar Epithelial were treated with control, HDL, LPS and HDL + LPS, and migration into the wound was photographed. **d** Quantification values are expressed as the mean ± SD (*n* = 3). (***P < 0.001)

HDL increased the migration of type II alveolar epithelial cells in the wound area by 76% vs. control and by 133% vs. LPS; HDL+ LPS also increased type II alveolar epithelial cells migration by 93% .vs. LPS. However, HDL+ LPS decrease type II alveolar epithelial cells migration to 17% compare to HDL. (Figure 2 b and d, *n* = 3; *p* < 0.001 comparing HDL vs. control, *p* < 0.001 for HDL+ LPS vs. LPS). In addition, to determine the effect of HDL to the migration of type II alveolar epithelial cells incubated with control media, LPS, HDL, or HDL+ LPS for 8 h. Transwell migration assay showed that HDL promoted the migration of type II alveolar epithelial cells, while LPS inhibited the migration. HDL increased the migration cell by 116% vs. control and by 208% vs. LPS; HDL+ LPS increased migration by 154% .vs. LPS. However, HDL+ LPS decreased migration to 18% compare to HDL. (Figure 2 a and c, *n* = 3; *p* < 0.001 comparing HDL vs. control, *p* < 0.001 for HDL+ LPS vs. LPS or HDL).

### HDL promotes type II alveolar epithelial cells adhesion to ECM

We observed significantly increased adhesion of type II alveolar epithelial cells to ECM after HDL pretreatment compared to control. In addition, HDL+ LPS statistically increased adhesion vs. LPS. (Figure 1 b, *n* = 3).

### HDL up-regulates the expression of ABCA1 in type II alveolar epithelial cells

**Fig. 3:**
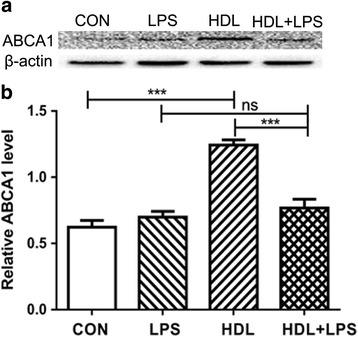
HDL Up-regulates the Expression of ABCA1. **a** Type II Alveolar Epithelial were treated with control, HDL, LPS and HDL + LPS, and ABCA1 expression was shown (**b**) (*n* = 3) (***P < 0.001; ns, not significant)

The apoA-I/ABCA1 pathway maintains normal lipid homeostasis in the lung [1]. Many studies have demonstrated a protective role for an apoA-I/ABCA1 pathway in the pathogenesis of lung disease [4]. The effect of epithelial migration and proliferation play an important role in the damage repairing of lung. To determine whether ABCA1, an important cholesterol transporter, was involved in functional of HDL, we examined the levels of cellular ABCA1 by Western blot. HDL led to 99% increased ABCA1 expression in type II alveolar epithelial cells compared to control. And it showed lower protein expression of ABCA1 after treated with LPS (Fig. 3 a and b).

### HDL activates signaling pathways

**Fig. 4:**
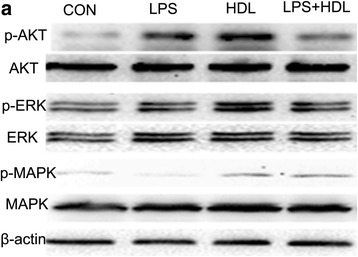
HDL Activates Signaling Pathways. **a** Type II Alveolar Epithelial were treated with control, HDL, LPS and HDL + LPS, and the signaling pathway of Akt, ERK and p38MAPK phosphorylation were tested by western blot (*n* = 3)

Akt, ERK and p38MAPK phosphorylation had been demonstrated to play a role in the signal transduction leading to endothelial cells proliferation and migration [13, 14]. Whether the similar signal pathways were seen in type II alveolar epithelial cells is unknown. Here cell signaling pathways in type II alveolar epithelial cells was performed to assess activation levels of Akt, ERK and p38MAPK pathways with HDL treatment as shown in Fig. 4. In this study, we test that HDL led to significant increase of AKT, ERK and p38MAPK phosphorylation in type II alveolar epithelial cells compared to control. In addition, HDL+ LPS increased the phosphorylation of p38MAPK vs. LPS. While the significant change didn’t seen in the phosphorylation of ERK and AKT.

### HDL inhibites secretion of TNF-a、IL-1a and IL-6 and the expression of CFTR

**Fig. 5:**
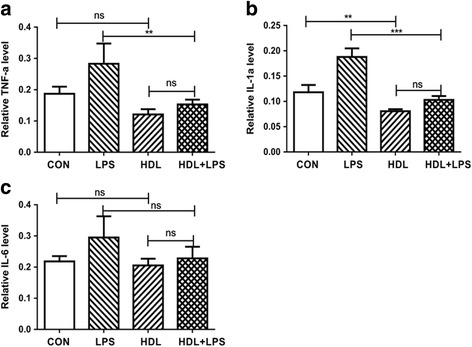
HDL Inhibits Secretion of TNF-a、IL-1a. Type II Alveolar Epithelial were treated with control, HDL, LPS and HDL + LPS. And TNF-a (**a**), IL-1a (**b**) and IL-6 (**c**) in media were measured by ELISA (*n* = 3) (**P < 0.01; ***P < 0.001; ns, not significant)

The secretion of proinflammatory factors play crucial role on development of inflammation of lung. Many studies had revealed that the proinflammatory factory like TNF-a, IL-1a and IL-6 have an effect on the lung disease. Our study founding that HDL significantly inhibited secretion of TNF-a, IL-1a but not IL-6. As shown in Fig. 5, LPS highly increased the secretion of TNF-a and IL-1a. HDL+ LPS decreased the secretion of TNF-a by 46% and IL-1a by 45% vs. LPS, but not exhibit statistical significance compare with HDL. In addition, there is no difference in the secretion of IL-6 among LPS group, HDL group and HDL+ LPS group seen in Fig. 5a-c.

**Fig. 6:**
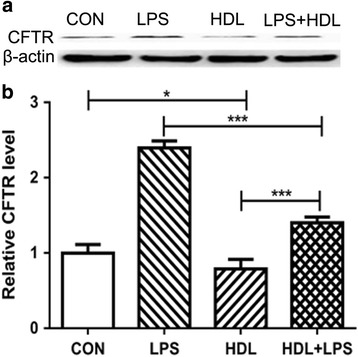
HDL Inhibits CFTR expression. **a** Alveolar Epithelial were treated with control, HDL, LPS and HDL + LPS, and CFTR expression was shown (**b**) (*n* = 3) (*P < 0.05; **P < 0.01; ***P < 0.001)

Furthermore, HDL treatment led to 25% decreased CFTR expression in type II alveolar epithelial cells compared to control treatment. And it showed higher protein expression of CFTR after treated with LPS seen in Fig. 6.

## Discussion

HDL play key roles in reverse cholesterol transport out of cells. Furthermore, HDL has anti-oxidative, anti-inflammatory, anti-apoptotic, and additional protective functions. These patterns are increasingly being identified to be active in the pulmonary system. HDL play an important protective role in normal lung health and in a variety of disease states as it modulates normal lung development and lung lipid homeostasis [4].

Ingrid et al. put forward that HDL is the primary source of vitamin E for type II pneumocytes and alveolar surfactant is supplemented with vitamin E during its assembly in type II pneumocytes [7]. Sharifov et al. test that apolipoprotein A-I (apoA- I, the major protein of) directly bind and neutralize LPS, which is derived from the cell walls of gram-negative bacteria, as well as lipoteichoic acid (a cell wall component of gram-positive bacteria) [15]. Wurfel et al. propose that apoA-I can also associate with the lipopolysaccharide-binding protein, which binds and neutralizes LPS [16]. In this study, we test that HDL stimulate type II alveolar epithelial cells proliferation and migration. As we known, alveolar epithelial cells (AEC) regulates gas exchange across the alveolo-capillary membrane and play a key role in keeping alveoli relatively ‘dry’ as it continuously carryout liquid from the alveolar space through the cationic and water channels located at the AEC apical surface [17]. Type II alveolar epithelial cells take charge of the production and turnover of pulmonary surfactant, the protein–lipid mixture that serves to lessen the surface tension in the alveolus permitting for lung ventilation smoothly^1^. Type II alveolar epithelial cells are responsible for epithelium reparation upon injury and ion transport [18]. Type II alveolar epithelial cells contribute also to lung defense by secreting antimicrobial products such as complement, lysozyme, and surfactant proteins. So the promotion of proliferation and migration of type II alveolar epithelial cells benefit the defense of lung disease.

As we known, ABCA1 is one of the major proteins involved in receptor-mediated cholesterol efflux. ABCA1 transporter is expressed by type I and type II alveolar epithelial cells, alveolar macrophages, pulmonary vascular endothelial cells (PVECs), and airway smooth muscle cells in the adult lung [19–21]. McNeish et al. [22] and Bates et al. [23] test that ABCA1 play an important role in maintaining normal lipid homeostasis in the lung as they finding alveolar macrophages and type II alveolar epithelial cells from *Abca1*-deficient mice are enriched with cholesterol. Bortnick et al. propose that ABCA1 expression in type II cells increases the removal of cholesterol mass and decreases basal rates of surfactant secretion [24]. In this study, we demonstrate that HDL treatment led to highly increased ABCA1 expression in type II alveolar epithelial cells. So we think that maybe HDL stimulate type II alveolar epithelial cells proliferation and migration by increasing the expression of ABCA1 in type II alveolar epithelial cells.

HDL has multiple endothelial actions which afford cardiovascular protection, among which, EC proliferation and migration may play a crucial role in vascular self-repair [25]. And many studies have test that HDL exerted its protective effects through stimulating several signaling pathways, including PI3K/Akt, Erk1/2 and p38MAPK activation in endothelial cell. As we known the effect of epithelial migration and proliferation play an important role in the damage repairing of lung. Here we experiment that the signaling pathways of PI3K/Akt, Erk1/2 and p38MAPK activation in epithelial cell. In this study, HDL treatment led to significant increasement phosphorylation of AKT, ERK and p38MAPK in type II alveolar epithelial cells vs control treatment. While during the inflammation state by LPS, HDL increased the phosphorylation of p38MAPK but not ERK and PI3K/Akt. All this indicate that the signaling pathways of PI3K/Akt, Erk1/2 and p38MAPK activation play an important role in normal lung health. And the signaling pathways of p38MAPK activation has a protective effect on inflammation of lung.

The secretion of proinflammatory factor is a crucial part in inflammation of lung. Kwon et al. and Sharifov et al. test that apoA-I avoided rodents from suffering neutrophilic airway inflammation and ALI in models of LPS- or LTA-mediated systemic inflammation. Xiao et al. experiment that administration of human HDL to mice has also attenuated LPS-induced acute lung injury (ALI) [26]. Sharifov et al. study that treatment of LPS-stimulated human blood with the L-4F apoA-I mimetic peptide suppressed endotoxin activity and IL-6 secretion [15]. Alveolar epithelial cells may promote to inflammatory events in ALI/acute respiratory distress sydrome (ARDS) and are an important source of cytokines (eg. TNF-a, IL-1b, IL-6) and chemokines (eg. monocyte chemotactic protein MCP-1, IL8) under inflammatory Conditions [27–29]. In this experiment, LPS highly increased the secretion of TNF-a and IL-1a but not IL-6. And HDL inhibit the releasing of TNF-a and IL-1a during LPS-stimulated inflammation state. CFTR, a cAMP dependent and ATP-gated chloride channel that regulates epithelial surface fluid secretion in respiratory, link to chronic lung inflammation [30]. This study revealed that HDL decrease the expression of CFTR in type II alveolar epithelial cells.

## Conclusion

HDL elevates the expression of ABCA1 and activates the signaling pathways of p38MAPK which promotes epithelial migration and proliferation during LPS-stimulated inflammation state. In addition, HDL decreases the secretion of TNF-a and IL-1a. In summary, our findings favor the notion that HDL plays a protective role in inflammation of lung, at least in part, as a result of promoting migration and proliferation of type II alveolar epithelial cells and inhibiting the releasing of proinflammatory factory like TNF-a and IL-1a.

## Abbreviations

ABCA1: ATP-binding cassette transporter A1
CFTR: Cystic fibrosis transmembrane conductance regulator
ERK: Extracellular signal-regulated kinase
HDL: High density lipoprotein;
IL: Interleukin
LPS: Lipopolysaccharide
MAPK: Mitogen-activated protein kinase
TNF: Tumor necrosis factor
